# Targeting Tristetraprolin Expression or Functional Activity Regulates Inflammatory Response Induced by MSU Crystals

**DOI:** 10.3389/fimmu.2021.675534

**Published:** 2021-07-16

**Authors:** Linxi lv, Ting Qin, Qiushi Huang, Hui Jiang, Feng Chen, Fan Long, Long Ren, Jianpin Liu, Yongen Xie, Mei Zeng

**Affiliations:** ^1^ Institute of Rheumatology and Immunology, Affiliated Hospital of North Sichuan Medical College, Nanchong, China; ^2^ Biology Group of Preclinical School of North SiChuan Medical College, Nanchong, China; ^3^ Clinical Lab of The Fifth People’s Hospital of Nanchong City, Nanchong, China; ^4^ Medical Imaging Key Laboratory of Sichuan, North SiChuan Medical College, Nanchong, China; ^5^ Academician (Expert) Workstation, Affiliated Hospital of North Sichuan Medical College, Nanchong, China

**Keywords:** TTP, Arctigenin, NLRP3 inflammasome, mitochondrial ROS, autophagic flux 3

## Abstract

The RNA-binding protein tristetraprolin (TTP) is an anti-inflammatory factor that prompts the mRNA decay of target mRNAs and is involved in inflammatory diseases such as rheumatoid arthritis (RA). TTP is regulated by phosphorylation, and protein phosphatase 2A (PP2A) can dephosphorylate TTP to activate its mRNA-degrading function. Some small molecules can enhance PP2A activation. Short interfering RNA (siRNA) targeting TTP expression or PP2A agonist (Arctigenin) was administered to monosodium urate (MSU) crystal-induced J774A.1 cells, and the expression of inflammatory related genes was detected by RT-PCR and Western blot assays. The effects of Arctigenin in mouse models of acute inflammation induced by MSU crystals, including peritonitis and arthritis, were evaluated. The data indicated that TTP expression levels and endogenous PP2A activity were increased in MSU-crystal treated J774A.1 cells. TTP knockdown exacerbated inflammation-related genes expression and NLRP3 inflammasome activation. However, PP2A agonist treatment (Arctigenin) suppressed MSU crystal-induced inflammation in J774A.1 cells. Arctigenin also relieved mitochondrial reactive oxygen species (mtROS) production and improved lysosomal membrane permeability in MSU crystal-treated J774A.1 cells. Moreover, TTP knockdown reversed the anti-inflammatory and antioxidant effects of Arctigenin. Oral administration of Arctigenin significantly alleviated foot pad swelling, the number of inflammatory cells in peritoneal lavage fluids and the production of IL-1β in the mouse model of inflammation induced by MSU crystals. Collectively, these data imply that targeting TTP expression or functional activity may provide a potential therapeutic strategy for inflammation caused by MSU crystals.

## Introduction

Gout is caused by the deposition of MSU crystals in and around the joints (1). Currently, gout is the most common cause of inflammatory arthritis and its global epidemiology shows an increase in incidence and prevalence in both developed and developing countries (2). Acute gout episodes are clinically described as arthritic pain and inflammation that, if left untreated, can develop into recurrent acute urate deposition (gout) and progressive joint destruction affecting the patient’s health (3, 4). However, to date, the clinical use of drugs has often resulted in undesirable side effects. Countless efforts have failed to create an effective and safe agent to treat gout.

Tristetraprolin (TTP, encoded by Ttp, also known as Zfp36) is one of the most characterized RNA binding proteins (RNA-BPs), and it mediates the instability of mRNA, recognizes ARE sequences through adjacent AUUUA-binding sites, and posttranscriptionally regulates the expression of tumor and inflammation-related genes (5). In the past decade, a large number of studies have shown that TTP plays an important role in balancing the inflammatory response (6) and plays a potential antitumor role by mediating the levels of proinflammatory cytokines such as IL-1β, TNF-α, IL-6 and COX-2 (7–9).

Some studies have revealed that TTP is regulated by p38 mitogen-activated protein kinase (MAPK). However, inhibiting the latter does not increase the level of unphosphorylated TTP (10, 11). Multiple inflammatory signaling pathways are regulated by PP2A, PP2A acts upstream of TTP signaling *in vitro* and *in vivo* (11, 12). PP2A activates TTP *via* dephosphorylation at S52 and S178, leading to the destabilization of target mRNAs (13). As agonist of PP2A, Arctigenin has anti-inflammatory effects (11, 12, 14). However, it is not clear whether TTP is involved in the inflammatory response induced by MSU crystals and whether PP2A activator can activate TTP and inhibit the inflammatory response induced by MSU crystals.

In this study, we found that TTP knockdown could aggravate the expression of inflammation-related genes induced by MSU crystals. However, Arctigenin, as a natural agonist of PP2A, can mitigate the inflammation induced by MSU crystals. The mechanisms may be involved in regulating mitochondrial function and oxidative stress as well as promoting autophagy flux, thereby inhibiting the activity of NLRP3 inflammasome in a TTP-dependent manner. More importantly, Arctigenin could protect against inflammatory models of gout in mice, such as peritonitis and arthritis.

## Materials and Methods

### Preparation of MSU Suspension

MSU crystals were prepared as previously described (15). One gram of uric acid (Sigma-Aldrich, St. Louis, MO, USA) was dissolved in 200 mL boiling water containing 1N NaOH, NaOH solution was added to adjust the pH to 8.9, and the solution crystallized overnight at room temperature. The precipitate was filtered out of the solution and dried at 42°C. The crystals were weighed under sterile conditions and suspended in PBS solution at a concentration of 25 mg/mL.

### Cell Culture and Transfection of siRNA

J774A.1 cells were cultured with DMEM containing 10% FBS. After the J774A.1 cells were transfected with scramble RNA (scRNA) or siRNA against the mouse TTP gene (siTTP) for 48 h, the cells were primed with LPS (100 ng/ml) for 1 h and subsequently challenged with the MSU suspension. Transfection of siRNA was performed using the INTERFERin siRNA Transfection Reagent following the manufacturer’s instructions. The siRNA sequences were synthesized by Sango Biotech (Shanghai, China). The target mouse TTP siRNA sequences were as follows: 5’-CGACAAAGCAUCAGCUUCUTT-3’ (sense) and 5’-AGAAGCUGAUGCUUUGUCGTT-3’ (antisense).

### ELISA

IL-1β/IL-6/TNF-a levels were detected by an ELISA kit according to the manufacturer’s instructions (NeoBioScience kit, Shenzhen, China).

### PP2A Activity Assay

PP2A activity was detected using the PP2A Immunoprecipitation Phosphatase Assay Kit (Merck Millipore, Darmstadt, Germany) following the manufacturer’s instructions.

### Extraction of Protein From Cell Culture Supernatants for Detecting Caspase-1 and IL-1β by Immunoblotting

Cell culture supernatants were collected and precipitated in deoxycholate containing 20% trichloroacetic acid (20% TCA) and washed three times with 100% acetone, before concentration in 1× Laemmli buffer.

### Western Blot Analysis

RIPA buffer was applied to extract total protein from J774A.1 cells or foot pad tissue. Nuclear protein was extracted using the CellLytic™ NuCLEAR™ Extraction Kit (Sigma, USA). The protein concentration was detected using a BCA protein assay kit (Thermo Scientific, MA, USA). The proteins were suspended in 1×Laemmli Buffer, boiled at 95°C for 6 minutes and separated by SDS-PAGE. The proteins were transferred to a PVDF membrane. The membrane was incubated with the primary antibodies at 4°C overnight. After washing the membrane, the membrane was incubated with HRP-conjugated secondary antibody (BA1054, dilution 1:7500, BosterBio, Wuhan, China) for 1 h at room temperature. Finally the membrane was exposed to the gel imaging system with a chemiluminescence kit and imaged with a gel imaging system. The density of protein bands was measured by ImageJ software. The ratio of target protein density to corresponding reference protein density is the relative expression of target gene in each group. The fold change of the treatment group was then calculated. Finally, statistical analysis was carried out. The details of the antibodies are in the **Supplementary File**.

### RNA Extraction and Quantitative Real-Time PCR

Total RNA was isolated from J774A.1 cells or foot pad tissue with TRIzol Reagent (TIANGEN, China), and cDNA was synthesized with a reverse transcriptase kit. A real-time system (Roche, USA) was used for PCR with SYBR Green Master Mix. The relative amounts of the target mRNA were normalized to the expression level of the reference gene GAPDH, and the data were analyzed through the 2^−ΔΔCt^ method. The primer sequences are shown in **Table 1**.

**Table 1:** The primers used for quantitative PCR.

Gene	Forward sequence (5′–3′)	Reverse sequence (5′–3′)
Mouse TTP	TCTCTGCCATCTACGAGAGCC	TCCTCCGAGGGATTCGGTTC
Mouse IL-1β	GAAATGCCACCTTTTGACAGTG	TGGATGCTCTCATCAGGACAG
Mouse IL-6	CTGCAAGAGACTTCCATCCAG	AGTGGTATAGACAGGTCTGTTGG
Mouse TNF-α	CCTGTAGCCCACGTCGTAG	GGGAGTAGACAAGGTACAACCC
Mouse COX-2	TGCACTATGGTTACAAAAGCTGG	TCAGGAAGCTCCTTATTTCCCTT
Mouse iNOS	GGAGTGACGGCAAACATGACT	TCGATGCACAACTGGGTGAAC
Mouse NLRP3	ATTACCCGCCCGAGAAAGG	CATGAGTGTGGCTAGATCCAAG
Mouse TTP	CCGAATCCCTCGGAGGACTT	GAGCCAAAGGTGCAAAACCA
Mouse Lamp1	CAGCACTCTTTGAGGTGAAAAAC	CCATTCGCAGTCTCGTAGGTG
Mouse Lamp2	TGTATTTGGCTAATGGCTCAGC	TATGGGCACAAGGAAGTTGTC
Mice Ctsb	CAGGCTGGACGCAACTTCTAC	TCACCGAACGCAACCCTTC
Mouse Ctsd	GCTTCCGGTCTTTGACAACCT	CACCAAGCATTAGTTCTCCTCC
Mouse SOD1	AACCAGTTGTGTTGTCAGGAC	CCACCATGTTTCTTAGAGTGAGG
Mouse SOD2	CAGACCTGCCTTACGACTATGG	CTCGGTGGCGTTGAGATTGTT
Mouse GPX1	CCACC GTGTA TGCCT TCTCC	AGAGAGACGCGACATTCTCAAT
Mouse CAT	TGGCACACTTTGACAGAGAGC	CCTTTGCCTTGGAGTATCTGG
Mouse GAPDH	AGGTCGGTGTGAACGGATTTG	GGGGTCGTTGATGGCAACA

### Detection of Total and Mitochondrial Reactive Oxygen Species

Mitochondrial superoxide indicator (Mito-SOX, Invitrogen; Thermo Fisher Scientific, Inc., USA) and Mito-Tracker Green (40742ES50) co-localization was used to detect mitochondrial ROS by Laser confocal microscopy imaging analysis following the described protocol (16). Cellular and mitochondrial ROS were respectively measured using the fluorescence probe DHE (BB-47-51, BestBio, China) and Mito-SOX fluorescence probe (Invitrogen; Thermo Fisher Scientific, Inc., USA) were detected by FACS following the manufacturer’s instructions. The mean fluorescence intensity (MFI) of PE-Texas Red-H was quantified using the FlowJo software.

### Mitochondrial Membrane Potential Detection

Mitochondrial membrane potential (MMP) was evaluated with JC-1 probe (Beyotime, Shanghai, China, C2006) following the manufacturer’s instructions. Briefly, J774A.1 cells were stained with JC-1 probe using the Olympus Laser confocal microscope imaging analysis or FACS measurement. In the FACS analysis, the ratio of PE-H (MFI)/FITC-H (MFI) was used to indicate MMP alternation through FlowJo software.

### LysoTracker and Acridine Orange Staining

Lysosome staining was respectively performed with acridine orange (Solarbio, Life Science) and Lysosome LysoTracker™ Deep Red (ThermoFisher Scientific, L12492) following the manufacturer’s instructions, and then capture the image and analyze fluorescence intensity using Laser confocal microscope.

### Determination of Intracellular Antioxidant Enzyme Activity

J774A.1 cells were washed with cold PBS for three times, and lysis buffer (20 mM Tris buffer pH 7.5, 150 mM NaCl and 1% Triton X-100) at 4°C for 15 min. The cell lysates were centrifuged, and the supernatants were collected. The activities of SOD, GSH-PX, and CAT were tested using the corresponding assay kits (Beyotime, China) according to the manufacturer’s instructions. The protein content was measured by a BCA assay kit (Thermo Fisher Scientific) following the manufacturer’s protocol, and the data are expressed as U/mg protein.

### Mice

Male C57BL/6 mice aged 6 to 8 weeks were purchased from the Dossy Experimental Animals Company (Chengdu, China). All animal experiments were conducted in accordance with the Guidelines for the Care and Use of Experimental Animals and were approved by the Ethics Committee of North Sichuan Medical College.

### MSU Crystals Induced Peritonitis and Arthritis Mice Model

To induce peritonitis, 1 h after oral ATG (Cayman Chemicals, #14913) administration 40 mg/kg; ATG dissolved in 5% DMSO, vehicle-treated mice were administrated orally 5% DMSO), mice were intraperitoneally injected with the MSU suspension (3 mg in 200 ml sterile PBS). Six hours after injection of MSU suspension, the mice were euthanized and peritoneal cavities were washed with 10 ml PBS. Peritoneal lavage fluids were analyzed for IL-1β production by ELISA and for lymphocyte recruitment by FACS using the leukocyte common antigen FITC-CD45, the macrophage marker PE-F4/80, the neutrophil marker APC-(Ly-6G and Ly-6C), basophilic granulocyte marker APC-CD63. FITC-CD45 (553079), PE-F4/80 (565410) and APC-(Ly-6G and Ly-6C) (553129) were obtained from BD BioScience. APC-CD63 (143906) was purchased from Biolegend.

The arthritis mouse model was given ATG orally. 1 h later, 1 mg of MSU (in 40 μl of PBS) was injected into the right foot pad and the same volume of PBS was injected into the left foot pad as the control. 24 h after injection of MSU suspension, the swelling of the foot pad was measured, and then the mice were sacrificed. Joint index evaluation was based on a method described previously (15). Part of the foot pad tissue was added with RIPA buffer to extract protein, and the rest of the foot pad tissue was fixed and sliced for hematoxylin and eosin (H&E) staining and immunofluorescence detection. The details of the antibodies are in the **Supplementary File**.

### Statistical Analysis

GraphPad Prism 8 software was used for statistical analysis. The data were expressed as mean ± SEM. Statistical analysis was performed using One-way ANOVA analysis of variance. P < 0.05 was regarded as significantly different by using LSD or Dunnett’s T3 test.

## Results

### MSU Crystals Stimulated the Expression of Tristetraprolin *In Vitro* and *In Vivo*

**Figure 1:**
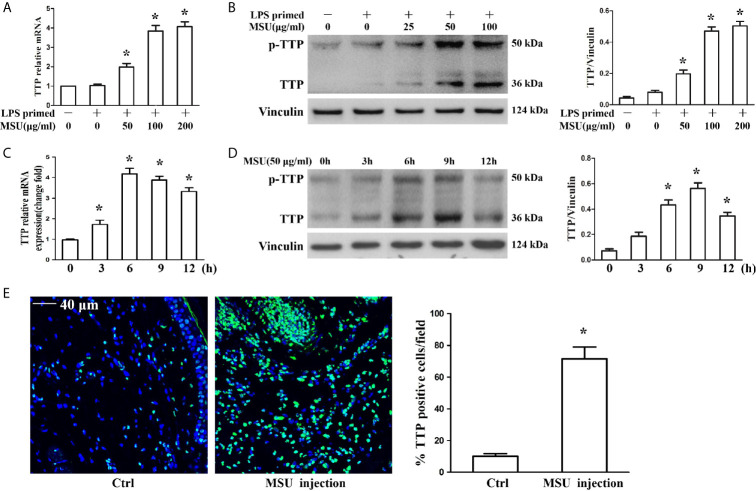
MSU crystals upregulated the expression of TTP *in vitro* and *in vivo*. **(A, B)** J774A.1 cells were primed with 100 ng/ml LPS for 1 h and then treated with different concentrations of MSU crystals for 12 h. **(A)** Total RNA was extracted from J774A.1 cells and reverse-transcribed, and the mRNA level of TTP was measured by qRT-PCR. *P < 0.05 *vs.* without MSU crystals treatment. **(B)** Total protein was isolated from J774A.1 cells, and TTP protein level was detected by Western blot. *P < 0.05 *vs.* without MSU crystals treatment. **(C, D)** J774A.1 cells were primed with 100 ng/ml LPS and then treated with 50 μg/ml MSU crystals at various time points (0, 3, 6, 9, 12 h). **(C)** Total RNA was extracted from J774A.1 cells, reverse-transcribed, and the mRNA level of TTP was measured by qRT-PCR. *P < 0.05 *vs.* without MSU crystals treatment. **(D)** Total protein was isolated from J774A.1 cells, TTP protein level was detected by Western blot. *P < 0.05 *vs.* without MSU crystals treatment. **(E)** Immunofluorescence assay was used to detect TTP protein distribution in mouse foot pad tissue sections. Blue shows nuclei stained with Hoechst 33342. Scale bar: 40 μm. (n=4 per group, mean ± SEM), *P < 0.05 *vs.* without MSU crystals injection in the foot pad tissue. All the data are expressed as the means ± SEM from n=3 independent experiments.

The murine macrophages cell line J774A.1 has been widely used as a cell model to study the activation of NLRP3 inflammasomes due to its expression of NLRP3, Caspase-1 and ASC (17, 18). To determine the influence of MSU crystals on TTP expression, J774A.1 cells were exposed to different doses of MSU crystals (25, 50 and 100 μg/ml) for 12 h, and the mRNA and protein levels of TTP were assessed. The quantitative RT-PCR and Western blot analysis data showed that MSU crystals increased TTP mRNA and protein expression in an almost dose-dependent manner (**Figures 1A, B**). Next, we sought to do a time course to assess the kinetics of MSU crystal-mediated TTP mRNA and protein levels over time. Cells were exposed to 50 μg/ml MSU crystals for the indicated time periods (0, 3, 6, 9 and 12 h), and after cell lysis, RNA and protein were extracted for quantitative RT-PCR and Western blot detection, respectively. MSU crystals significantly enhanced TTP mRNA expression with a peak at 6 h (**Figure 1C**), but TTP protein expression reached its peak when the J774A.1 cells were stimulated with 50 μg/ml MSU crystals for 9 h (**Figure 1D**). Based on the established mouse model of gouty arthritis, the immunofluorescence data of mouse foot pad tissue sections showed that the number of TTP positive cells was significantly increased in the MSU crystal-treated group (**Figure 1E**), suggesting that TTP might be involved in MSU crystal-induced gouty arthritis.

### Blocking Tristetraprolin Expression Accelerates the MSU Crystal-Induced Inflammatory Response in J774A.1 Cells

**Figure 2:**
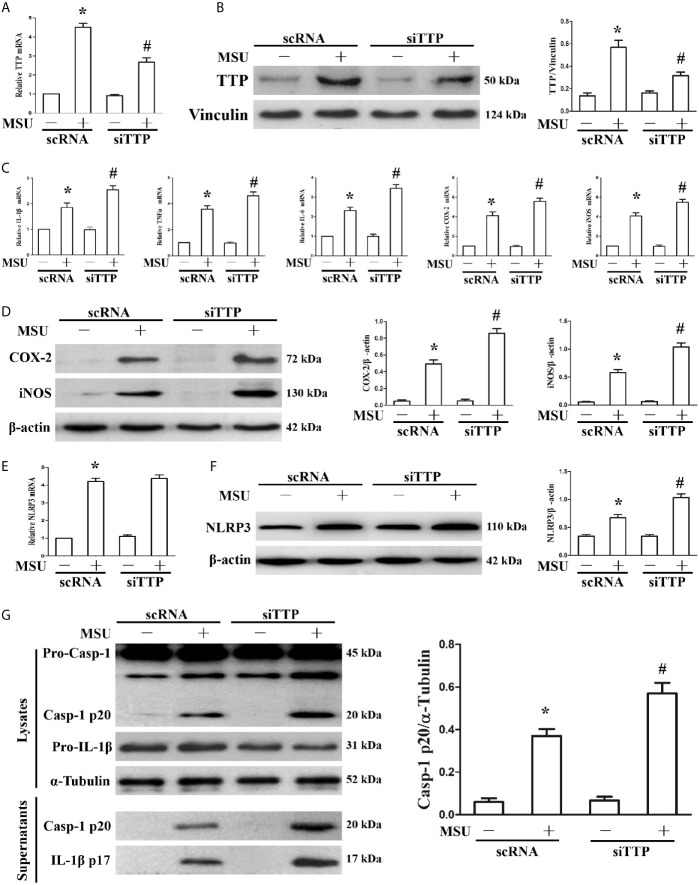
Influence of blocking TTP expression on the MSU crystal-induced inflammatory response in J774A.1 cells. **(A, B)** J774A.1 cells were transfected with scramble RNA (scRNA) or TTP-targeted siRNA (siTTP) for 48 h, primed with 100 ng/ml LPS and then stimulated with MSU crystals (50μg/ml) for 9 h. The knockdown of TTP mRNA and protein expression was confirmed by **(A)** RT-PCR and **(B)** Western blot (compared to α-tubulin as a loading control). **(C)** IL-1β, TNF-α, IL-6, COX-2 and iNOS mRNA expression was measured. **(D)** Western blot analysis of COX-2 and iNOS protein levels. **(E)** Effect of TTP knockdown on the NLRP3 mRNA level. **(F)** Impact of TTP knockdown on the NLRP3 protein level. **(G)** Effect of TTP knockdown on the protein levels of the p20 subunit of Caspase-1 and IL-1β in the cell lysates and culture supernatants. All the data are expressed as the means ± SEM from n=3 independent experiments, *P < 0.05 *vs.* without MSU crystals treatment; ^#^P < 0.05 *vs.* scRNA transfection + MSU crystals treatment.

We have found that MSU crystals can stimulate the expression of TTP. We further sought to demonstrate the effect of TTP knockdown on the expression of MSU crystal-induced inflammation-related gene. J774A.1 cells were transfected with TTP-targeted siRNA (siTTP) or scramble RNA (scRNA) and then stimulated with MSU crystals. As shown in **Figure 2A**, siRNA against TTP significantly alleviated MSU crystal-induced TTP mRNA expression. MSU crystal-induced TTP protein levels were similarly inhibited by siRNA against TTP, as detected by Western blot (**Figure 2B**). Transient primed LPS had little effect on the expression of IL-1β at the mRNA level (**Supplementary Figure 1**). Importantly, when TTP expression was inhibited, there was a corresponding increase in the mRNA expression of IL-1β, TNF-α, IL-6, COX-2 and iNOS in the MSU crystal-treated J774A.1 cells **(** **Figure 2C**). ELISA data also showed that after MSU crystals stimulation, the release of IL-1β, TNF-α and IL-6 in the culture supernatants from J774A.1 cells was upregulated, while these cytokines were further increased in MSU crystal-stimulated J774A.1 cells when TTP expression was inhibited (**Supplementary Figure 2**). In line with the RT-PCR data, Western blot analysis also revealed that the protein levels of COX-2 and iNOS in J774A.1 cells were enhanced after exposure to MSU crystals, and TTP knockdown further increased the iNOS and COX-2 protein levels in the MSU crystals stimulated J774A.1 cells **(** **Figure 2D**).

It has been reported that TTP can inhibit NLRP3 expression and NLRP3 inflammasome activation in LPS induced inflammation (19). The mRNA level of NLRP3 induced by MSU crystals remained almost unchanged by TTP knockdown (**Figure 2E**), but the MSU crystal treatment led to increased protein expression of NLRP3 (**Figure 2F**), suggesting that TTP mainly affected the translation of NLRP3 in the MSU crystals treated J774A.1 cells. Because TTP knockdown affected NLRP3 protein levels, we next determined whether it influenced the activation of the NLRP3 inflammasome. As expected, activation of the NLRP3 inflammasome in MSU crystal-induced J774A.1 cells resulted in the cleavage of Caspase-1 and IL-1β, and their release into the culture supernatant. Importantly, in TTP-specific siRNA transfected and MSU crystal-treated J774A.1 cells, the protein levels of both Caspase-1 and IL-1β secreted into the supernatants were elevated (**Figure 2G**).

### PP2A Agonist Attenuated MSU Crystal-Induced Inflammation in J774A.1 Cells

**Figure 3:**
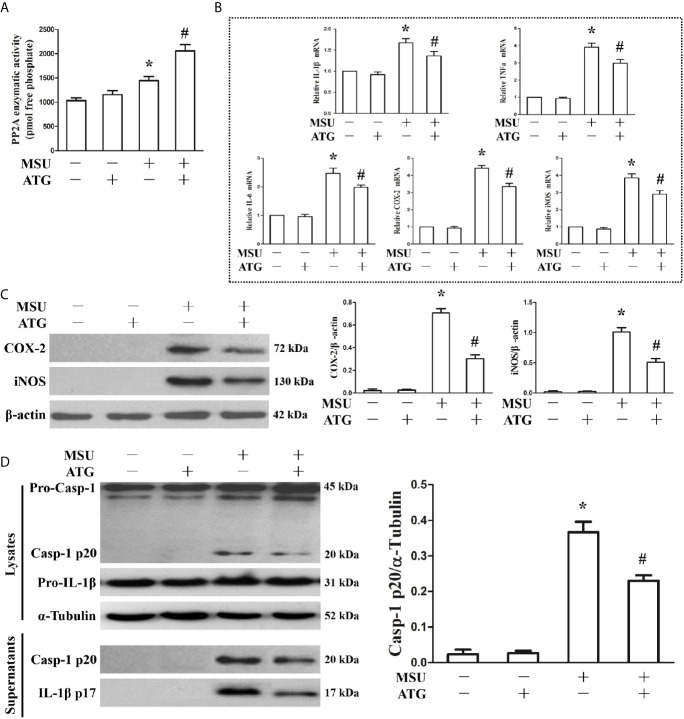
Effects of PP2A agonist (ATG) on PP2A phosphate activity and inflammation-related gene expression in MSU crystals stimulated J774A.1 cells. **(A–D)** J774A.1 cells were pretreated with ATG (5 μM) for 1 h, primed with LPS (100 ng/ml) for 1 h and then treated with MSU crystals (50 μg/ml) for 9 h. **(A)** Influence of a PP2A agonist (ATG) on MSU crystal-induced PP2A phosphatase activity. **(B)** The mRNA levels of IL-1β, TNF-α, IL-6, COX-2 and iNOS were detected using RT-PCR because of ATG treatment. **(C)** Western blotting was used to detect COX-2 and iNOS protein levels after ATG treatment. **(D)** The protein levels of the p20 subunit of Caspase-1 and IL-1β in the cell lysates and culture supernatants were measured using Western blot. All the data are expressed as the means ± SEM from n=3 independent experiments, *P < 0.05 *vs.* without MSU crystals treatment; ^#^P < 0.05 *vs.* MSU crystals treatment + vehicle.

Because PP2A phosphatase activity is closely related to the anti-inflammatory function of TTP, we sought to investigate the effect of MSU crystals on PP2A phosphatase activity in J774A.1 cells. As shown in **Figure 3A**, when J774A.1 cells were treated with MSU crystals, PP2A phosphatase activity significantly increased, implying that PP2A activity may be related to inflammation induced by MSU crystals. Recently, it has been confirmed that Arctigenin can increase PP2A activation (14). We sought to determine the effect of Arctigenin on PP2A activity in J774A.1 cells stimulated by MSU crystals. Arctigenin also greatly augmented PP2A activity induced by MSU crystals (**Figure 3A**). We further studied the effect of Arctigenin on the MSU crystal-induced expression of inflammation-related genes. Arctigenin relieved the mRNA expression of inflammation-related genes induced by MSU crystals (**Figure 3B**). Consistent with the RT-PCR data, treatment with Arctigenin suppressed the secretion of IL-1β, TNF-α and IL-6 in culture supernatants from in J774A.1 cells **(** **Supplementary Figure 3**).Western blot data revealed that Arctigenin treatment significantly reduced the protein levels of COX-2 and iNOS induced by MSU crystals in J774A.1 cells (**Figure 3C**). Meanwhile, the production of Caspase-1 and IL-1β in culture supernatants was also suppressed (**Figure 3D**), suggesting that suppressed the activity of the NLRP3 inflammasome *in vitro*. All these data imply that Arctigenin can inhibit the inflammatory response induced by MSU crystals *in vitro*.

### Tristetraprolin Knockdown Abrogates the Arctigenin-Mediated Inhibition of the MSU Crystal-Induced Inflammatory Response

**Figure 4:**
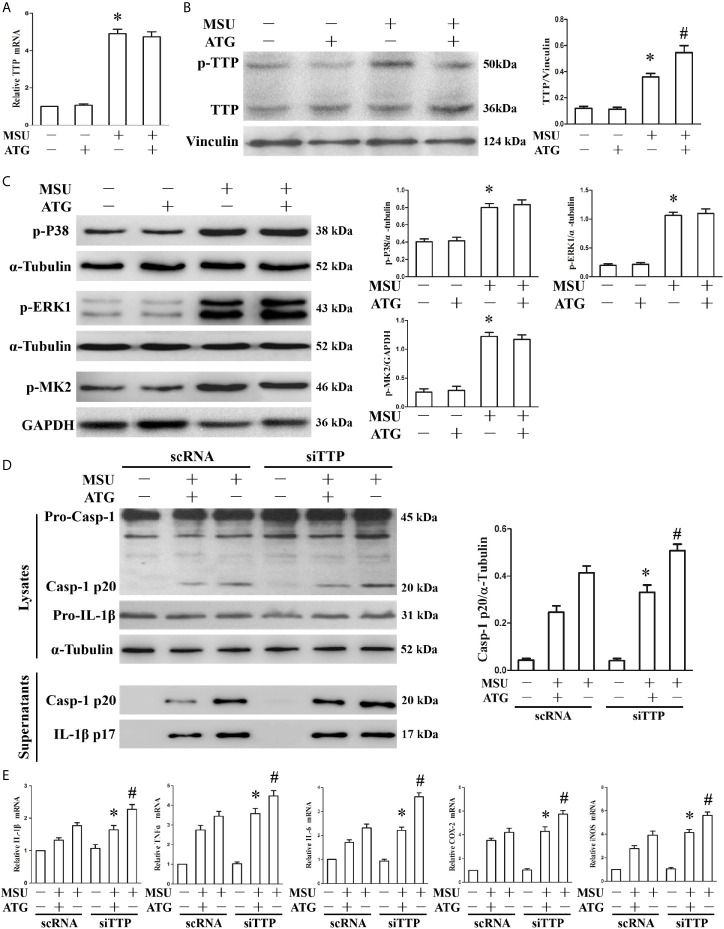
The effects of ATG on TTP expression and TTP knockdown abolishes the ATG-mediated repression of the MSU crystal-induced inflammatory response. **(A–C)** J774A.1 cells were pretreated with ATG (5 μM) for 1 h, primed with LPS for 1 h and then treated with MSU crystals (50 μg/ml) for 9 h. Total RNA and protein in the cells were extracted. *P < 0.05 vs. without MSU crystals treatment; ^#^P < 0.05 *vs.* MSU crystals treatment + vehicle. **(A)** The mRNA expression level of TTP was detected using RT-PCR. **(B)** Western blotting was used to measure the protein level of TTP. **(C)** The phosphorylation levels of MAPK p38, ERK1 and MK2 were tested using Western blot assays. **(D, E)** J774A.1 cells were either transfected with scRNA or siTTP for 48 h, treated with 5 μM ATG for 1 h, primed with LPS for 1 h, and then stimulated with MSU crystals (50 μg/ml) for 9 h. **(D)** The protein levels of the p20 subunit of Caspase-1 and IL-1β in the cell lysates and culture supernatants were measured using Western blot. All the data are expressed as the means ± SEM from n=3 independent experiments, *P < 0.05 *vs.* MSU crystals treatment + ATG + scRNA; ^#^P < 0.05 *vs.* MSU crystals treatment + scRNA. **(E)** The mRNA levels of IL-1β, IL-6, TNF-α, COX-2 and iNOS were determined. *P < 0.05 *vs.* MSU crystals treatment + ATG + scRNA; ^#^P < 0.05 *vs.* MSU crystals treatment + scRNA. All the data are expressed as the means ± SEM from n=3 independent experiments.

Thus far, we have found that TTP can regulate inflammation induced by MSU crystals, and Arctigenin can regulate PP2A activity and attenuate MSU crystal induced inflammatory gene expression *in vitro*. We hypothesized that Arctigenin might regulate its anti-inflammatory effects in a TTP-dependent manner. To confirm this link, we investigated the effect of Arctigenin on TTP expression. As shown in **Figure 4A**, MSU crystals significantly augmented TTP mRNA expression; however, this effect was unaffected by pretreatment with Arctigenin. We then examined the effect of Arctigenin on TTP protein levels. Because TTP protein has anti-inflammatory function when it is dephosphorylated, we speculate that Arctigenin may promote the level of dephosphorylated TTP protein. This was exactly what was observed in **Figure 4B**. Previous study indicated that phosphorylation of ERK and MAPK p38 in macrophages was significantly increased after MSU crystal stimulation (20). MAPK p38, when activated, in turn activates the downstream kinase MAPK activated protein kinase 2 (MK2). MK2 phosphorylates 52 and 178 serine of mouse TTP. ATG treatment did not affect extracellular signal-regulated kinase (ERK1), MAPK p38 or MK2 phosphorylation in response to MSU crystals (**Figure 4C**). Therefore, we speculated that the anti-inflammatory effects of ATG *in vitro* might depend on its ability to modulate the TTP phosphorylation state but do not involve the impairment of signaling events upstream of TTP phosphorylation. To confirm the relationship between PP2A activation and TTP-mediated expression of inflammation-related gene, we explored whether Arctigenin-mediated inhibition depended on TTP by knocking down TTP and observing the effect on the expression of inflammation-related gene. TTP knockdown reversed the ATG-mediated inhibition of NLRP3 inflammasome activity (**Figure 4D**) and inflammation-related genes at mRNA expression levels (**Figure 4E**). Consistent with the RT-PCR data, the ELISA data also indicated that specific knockdown of TTP with siRNA abrogated the suppression of IL-1β, TNF-α and IL-6 protein secretion by Arctigenin **(** **Supplementary Figure 4**). These data further support our hypothesis that PP2A activators may mediate their anti-inflammatory effects in a TTP-dependent manner.

### Arctigenin Ameliorates Mitochondrial Dysfunction Induced by MSU Crystals

**Figure 5:**
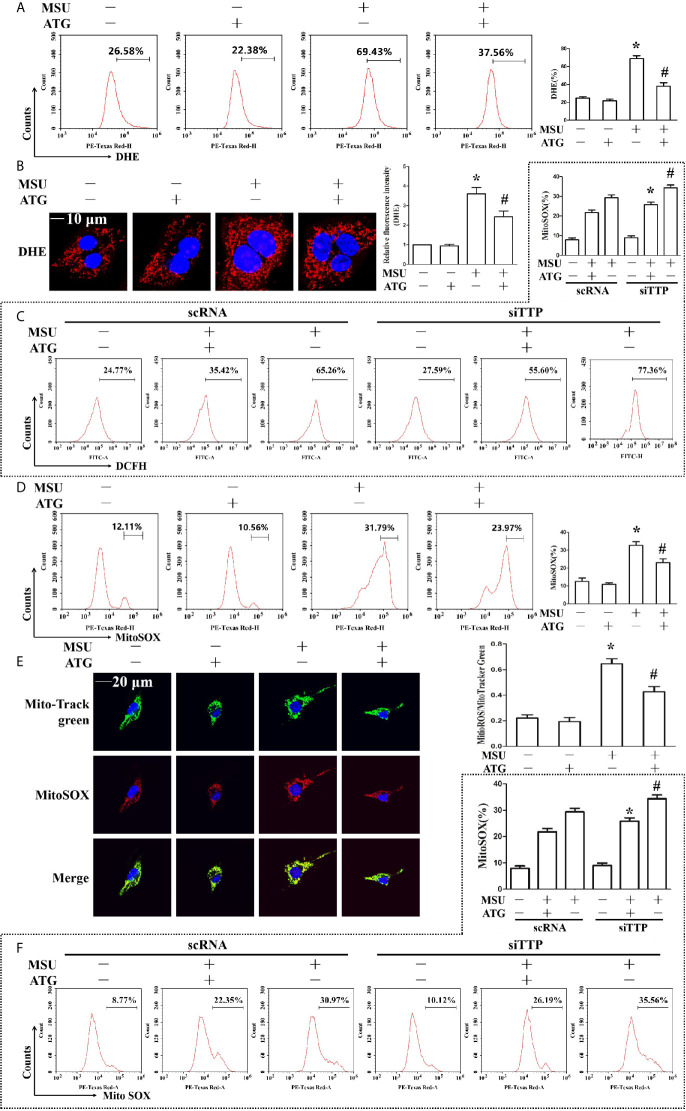
Effects of ATG administration on the MSU crystal-induced production of intracellular total ROS, mitochondrial ROS and mitochondrial membrane potential. **(A, B, D, E)** J774A.1 cells were pretreated with ATG (5 μM) for 1 h, primed with LPS for 1 h and then treated with MSU crystals (50 μg/ml) for 9 h. *P < 0.05 *vs.* without MSU crystals treatment; ^#^P < 0.05 *vs.* MSU crystals treatment +vehicle. **(C, F)** J774A.1 cells were either transfected with scRNA or siTTP for 48 h, treated with 5 μM ATG for 1 h, primed with LPS for 1 h, and then stimulated with MSU crystals (50 μg/ml) for 9 h. *P < 0.05 *vs.* MSU crystals treatment + ATG + scRNA; ^#^P < 0.05 *vs.* MSU crystals treatment + scRNA. **(A, B)** Cells were stained with DHE fluorescent probe, then the fluorescence intensity of DHE was measured by FACS or images were captured by Laser confocal microscope. Scale bar: 10 μm. **(C)** The fluorescence intensity of DCFH was measured by FACS. **(D)** Cells were stained with MitoSOX and then FACS was used to detect the fluorescence intensity of MitoSOX. **(E)** Cells were stained with MitoSOX and Hoechst 33342. Laser confocal microscope is used for image capture and fluorescence intensity analysis. Blue shows nuclei stained with Hoechst 33342. Scale bar: 20 μm. **(F)** FACS was used to detect the fluorescence intensity of MitoSOX. All the data are expressed as the means ± SEM from n=3 independent experiments.

**Figure 6:**
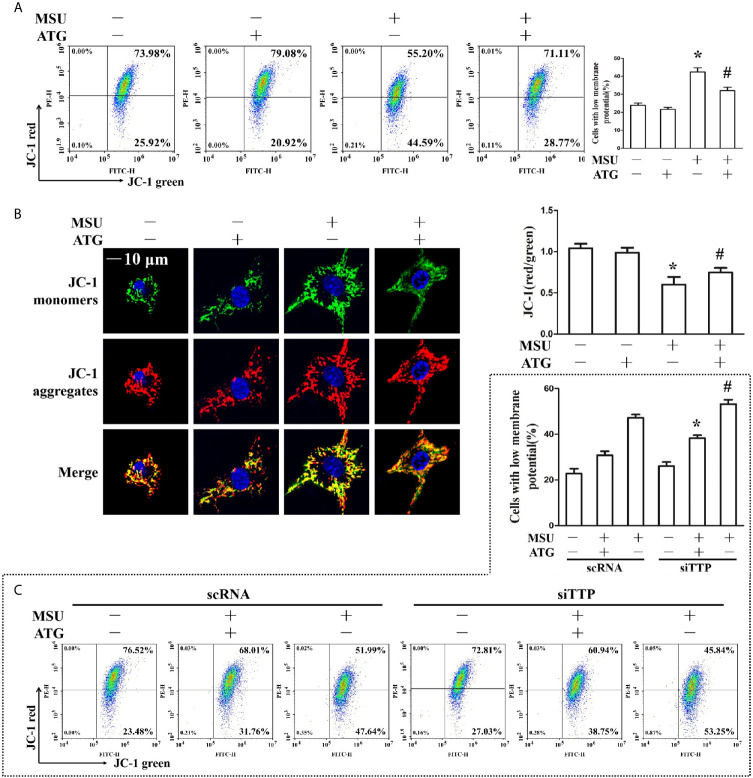
**(A, B)** J774A.1 cells were pretreated with ATG (5 μM) for 1 h, primed with LPS for 1 h and then treated with MSU crystals (50 μg/ml) for 9 h. *P < 0.05 *vs.* without MSU crystals treatment; ^#^P < 0.05 *vs.* MSU crystals treatment +vehicle. **(A)** Cells were stained with JC-1 probe and FACS was used to measure mitochondrial membrane potential. **(B)** Cells were stained with JC-1 probe and Hoechst 33342. Laser confocal microscope was used to capture the fluorescence image and analyze fluorescence intensity. Blue shows nuclei stained with Hoechst 33342. Scale bar: 10 μm. **(C)** J774A.1 cells were either transfected with scRNA or siTTP for 48 h, treated with 5 μM ATG for 1 h, primed with LPS for 1 h, and then stimulated with MSU crystals (50 μg/ml) for 9 h. Cells were stained with JC-1 probe and FACS was used to measure mitochondrial membrane potential. *P < 0.05 *vs.* MSU crystals treatment + ATG + scRNA; ^#^P < 0.05 *vs.* MSU crystals treatment + scRNA. All the data are expressed as the means ± SEM from n=3 independent experiments.

The involvement of ROS in the pathological process of acute gout has gained increasing recognition; moreover, it has been reported that Arctigenin has antioxidant functions (21). Hence, we explored the effect of Arctigenin on MSU crystal-induced oxidative stress. As shown in **Figures 5A, B**, we found that intracellular total ROS generation in the MSU crystal-treated J774A.1 cells increased up to almost 3-fold compared to that in the control cells. However, Arctigenin treatment dramatically attenuated the effect of MSU crystals on ROS generation. TTP knockdown reversed the regulation of ATG on the intracellular total ROS generation (**Figure 5C**). Intracellular ROS are mainly derived from mitochondria, so we further investigated the effect of ATG on mitochondrial ROS through mitochondrial superoxide indicators. The increase in mitochondrial ROS triggered by MSU crystals was blocked by Arctigenin treatment (**Figures 5D, E**). The regulation of ATG on mitochondria ROS was affected in TTP knockdown macrophages (**Figure 5F**). It has been reported that elevated mitochondrial ROS can damage mitochondria. To evaluate the level of mitochondrial damage, the mitochondrial membrane potential (ΔΨm) was detected using the fluorescent probe JC-1. MSU crystals significantly depolarized the mitochondrial membrane in J774A.1 cells, as shown by the shift in JC-1 fluorescence from red to green. Intervention with Arctigenin greatly relieved the damage of mitochondrial membrane potential caused by MSU crystals (**Figures 6A, B**). However, the improvement of mitochondrial membrane potential by ATG was impeded in TTP knockdown macrophages (**Figure 6C**). These data suggest that ATG can ameliorate the mitochondrial dysfunction induced by MSU crystals in a TTP-dependent manner.

To further elucidate the antioxidant effects of Arctigenin in MSU crystal-stimulated J774A.1 cells, we investigated the effects of Arctigenin on the activity and expression level of intracellular antioxidant enzymes. The activities of SOD, GPX and CAT in MSU crystal-treated cells were significantly lower than those in the control (**Supplementary Figure 5A**). However, Arctigenin treatment significantly relieved the MSU crystal-induced decrease in antioxidant enzymes activities (**Supplementary Figure 5B**). The mRNA expression levels of antioxidant defense-related genes, including SOD1, SOD2, GPX1 and CAT were also detected. A significant decrease in the mRNA expression of SOD1, SOD2, GPX1 and CAT was observed in the MSU crystal-treated cells compared with the control group. Arctigenin treatment effectively alleviated the decrease in the mRNA expression levels of SOD1, SOD2 and CAT caused by MSU crystals but had little effect on GPX1 expression (**Supplementary Figure 5B**). These data suggest that Arctigenin treatment can prevent the imbalance in antioxidant defense system of J774A.1 cell caused by MSU crystals.

### Arctigenin Inhibits Lysosomal Rupture, Accelerates Lysosomal Biogenesis and Facilitates Autophagic Flux

**Figure 7:**
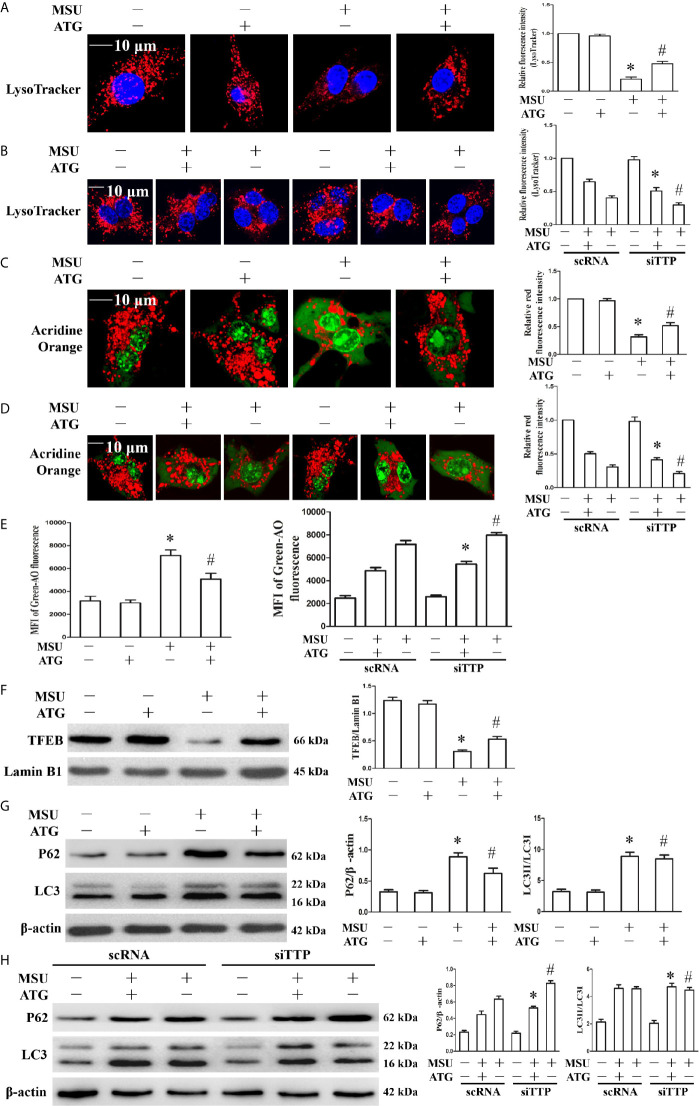
Effects of ATG treatment or TTP knockdown on MSU crystal-induced lysosomal rupture, lysosomal biogenesis and autophagic flux in J774A.1 cells. **(A, C, E–G)** J774A.1 cells were pretreated with ATG (5 μM) for 1 h, primed with LPS for 1 h and then treated with MSU crystals (50 μg/ml) for 9 h. *P < 0.05 *vs.* without MSU crystals treatment; ^#^P < 0.05 *vs.* MSU crystals treatment +vehicle. **(B, D, H)** J774A.1 cells were either transfected with scRNA or siTTP for 48 h, treated for 1 h with 5 μM of ATG, primed with LPS for 1 h, then stimulated with MSU crystals (50 μg/ml) for 9 h. **(A, B)** The cells were stained using LysoTracker Red (100 nM, 30 min) and then with Hochest 33342 (20 μg/ml, 15 min). Images were captured by Laser confocal microscope. Scale bar: 10 μm. **(C–E)** cells were incubated with AO for 30 min, and then images were captured by Laser confocal microscope or green fluorescence intensity of AO was determined by a microplate reader. Scale bar: 10 μm. **(F)** Western blot analysis of TFEB level in nuclear proteins. **(G, H)** Western blotting was used to measure the LC3II and P62 protein levels. The LC3II and P62 protein levels were measured by Western blot assay. *P < 0.05 *vs.* MSU crystals treatment + ATG + scRNA; ^#^P < 0.05 *vs.* MSU crystals treatment + scRNA. All the data are expressed as the means ± SEM from n=3 independent experiments.

Lysosomal dysfunction is closely associated with the activation of the NLRP3 inflammasome (22). It has been reported that lysosomal instability results in a decrease in the fluorescence intensity of the LysoTracker dye (23). In our study, MSU crystal-treated cells showed partial loss of the fluorescence signal of LysoTracker dye, while Arctigenin treatment abrogated the decrease in the fluorescence signal (**Figure 7A**). TTP knockdown hindered the regulation of ATG on lysosomal number (**Figure 7B**). Acridine orange produced red fluorescence when it accumulates within the lysosome and green fluorescence when it is released from ruptured lysosomes and diffuses into the cytoplasm and nucleus (24). Arctigenin treatment alleviated the decrease of red fluorescence signal induced by MSU crystals in the cytoplasm (**Figure 7C**), but in TTP knockdown cells, this function of ATG is disturbed (**Figure 7D**). Arctigenin treatment inhibited the increase in green fluorescence caused by MSU crystals (**Figure 7E**), TTP knockdown yet impeded this function of ATG (**Figure 7E**). These findings imply that ATG mitigates the MSU crystal-induced increase in lysosomal membrane permeability in a TTP-dependent manner.

TFEB (transcription factor EB) is a master transcription regulator of a subset of genes involved in lysosomal biogenesis and autophagy (25, 26). After MSU crystal treatment, the TFEB protein level in the nucleus was significantly reduced. Importantly, Arctigenin treatment counteracted the impairment of the TFEB protein level in the nucleus (**Figure 7F**). Next, we explored whether Arctigenin could induce lysosomal biogenesis, which is another potential reason for the increase in the red signal of LysoTracker staining, so we determined the effect of Arctigenin on the expression of lysosomal marker genes (Lamp1, Lamp2, Ctsb, and Ctsd). Quantitative RT-PCR data indicated that MSU crystal stimulation inhibited the mRNA expression of Lamp1 and Lamp2, and had little influence on the mRNA expression of Ctsb and Ctsd (**Supplementary Figure 6**). However, after Arctigenin pretreatment, Lamp1, Lamp2, Ctsb and Ctsd were all significantly elevated at the mRNA level (**Supplementary Figure 6**). Previous studies have shown that although MSU crystals can increase the expression of LC3-II, a marker for autophagosome formation, autophagic flux is blocked (20, 27). Arctigenin had no effect on the expression of LC3-II induced by MSU crystal, but significantly attenuated the expression of P62 induced by MSU crystals (**Figure 7G**). The results indicated that Arctigenin might promote autophagic flux and thus prevent p62 aggregation induced by MSU crystals. It has been confirmed that TTP plays an important role in autophagic flux and is necessary to form autophagolysomes and to clear pathogens (28). As shown in **Figure 7H**, by detecting LC3II and P62 protein levels, our data showed that Arctigenin-induced autophagic flux decreased in the TTP knockdown and MSU crystal-treated J774A.1 cells compared with the scRNA transfected and MSU crystal-treated J774A.1 cells. These results suggest that TTP is necessary for Arctigenin-mediated autophagic flux.

### Arctigenin Ameliorates MSU Crystal-Induced Peritoneal Inflammation and Arthritis *In Vivo*

**Figure 8:**
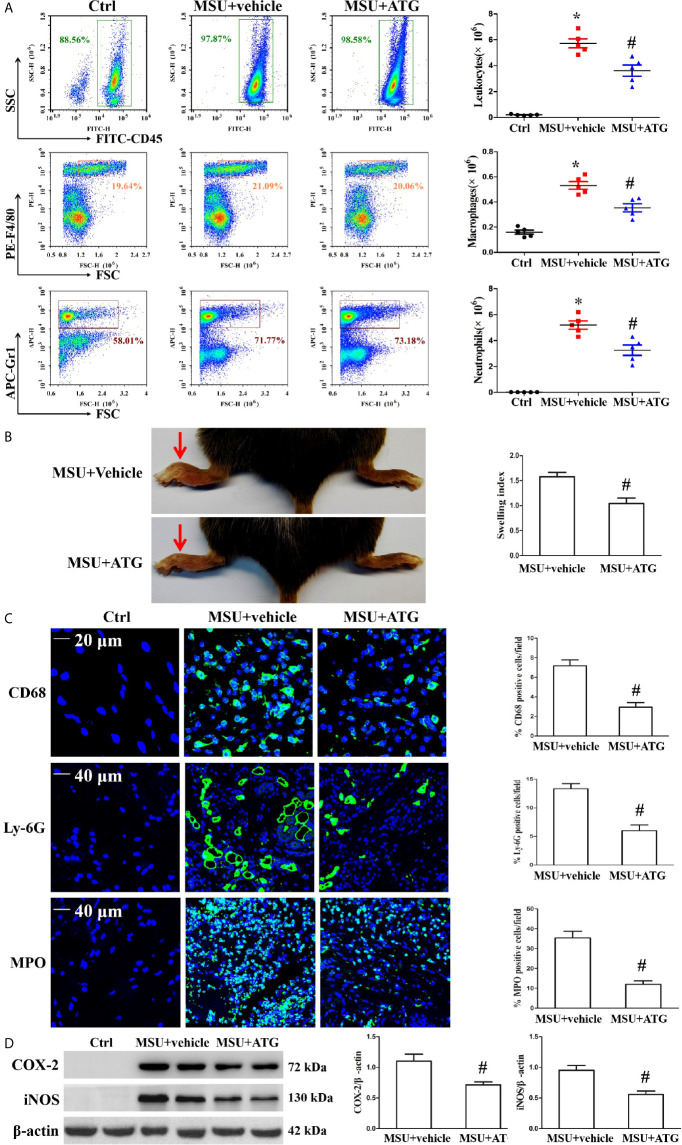
Effects of ATG on MSU crystal-induced inflammatory cell infiltration and cytokine release in a mouse model of peritonitis and arthritis. **(A)** After the cells precipitated from the peritoneal fluid and were respectively stained with FITC-CD45 Ab, PE-F4/80 Ab, and APC-(Ly-6G and Ly-6C) Ab. The percentage and cell numbers of migrated leukocytes, macrophages, and neutrophils were analyzed using FACS. **(B)** Paw swelling index. **(C)** An immunofluorescence assay was used to detect macrophage, neutrophil and MPO positive cells distribution in mouse foot pad tissue sections. Blue shows nuclei stained with Hoechst 33342. **(D)** Western blot assays were used to measure the protein levels of COX-2 and iNOS in foot pad tissue. n=5 for each group, *P < 0.05 *vs.* Ctrl; ^#^P < 0.05 *vs.* MSU crystals + vehicle. All the data are expressed as the means ± SEM from n=3 independent experiments.

MSU crystal deposition is the major cause of gouty arthritis. In the study, MSU crystals were respectively injected into the foot pad and into the abdominal cavity to induce arthritis and peritonitis to mimic the gout arthritis model. To confirm the role of Arctigenin in MSU crystal-induced inflammation *in vivo*, peritonitis model in C57BL/6 mice was used to evaluate the effect of Arctigenin on inflammatory cell influx and IL-1β production. The numbers of leukocytes, macrophages, neutrophils and basophilic granulocyte in the peritoneal fluid were assessed by staining the cells for the leukocyte marker CD45, the macrophage marker F4/80, the neutrophil marker Gr-1 and basophilic granulocyte marker CD63. As shown in **Figure 8A**, MSU crystals facilitated the infiltration of leukocytes, macrophages and neutrophils into the peritoneal cavity, but this was greatly suppressed by Arctigenin treatment (**Figure 8A**), there was little effect on the number of basophilic granulocytes (**Supplementary Figure 7**. Compared to vehicle treatment, Arctigenin treatment also greatly abrogated IL-1β secretion in peritoneal lavage fluids induced by MSU crystals (**Supplementary Figure 8A**).

Injection of MSU suspensions into the foot pad of mice can cause inflammation. Our data revealed that Arctigenin treatment relieved MSU crystal-induced swelling of the foot pad (**Figure 8B**). We measured IL-1β mRNA levels in foot pad tissue lysates and observed that Arctigenin dramatically blocked the expression of IL-1β (**Supplementary Figure 8B**). Histological analysis displayed obvious inflammatory cell infiltration in the sections of the foot pad tissue injected with MSU suspension (**Supplementary Figure 8C**), but Arctigenin administration visibly reduced the number of infiltrated leukocytes. We further confirmed the effect of Arctigenin administration on MPO positive cell distribution, Ly-6G^+^ neutrophil and CD68^+^ macrophage infiltration in foot pad tissue sections through immunofluorescence. The results showed that Arctigenin treatment prevented both neutrophils and macrophages from infiltrating into the foot pad tissue injected with the MSU suspension (**Figure 8C**) and blocked the protein levels of COX-2 and iNOS in the foot pad tissue injected with the MSU suspension (**Figure 8D**). Thus, these findings suggest that Arctigenin may be a potential candidate for the treatment of gouty arthritis.

## Discussion

It has been reported that TTP is a negative regulator of many proinflammatory factors and is strongly expressed in active inflammatory sites, including RA synovial lining cells (29, 30). The major targets of TTP are the mRNA transcripts of cytokines. The TTP protein exists in two forms, the phosphorylated form, which is inactive, and the unphosphorylated form, which is active and induces mRNA decay. Thus, when TTP is phosphorylated, cytokine expression is upregulated, but when TTP is unphosphorylated, the production of target cytokines is inhibited. Unphosphorylated TTP is less stable and is easily degraded by the UPS (31, 32). Moreover, several studies have revealed that PP2A agonists may promote an increase in anti-inflammatory TTP activity (11, 12, 33). All of these findings prompted us to explore the effects of targeting TTP expression or promoting PP2A functional activity on the MSU crystal-induced inflammatory response.

In this study, we showed that exposure to MSU crystals accelerated the expression of TTP both *in vitro* and *in vivo*, but we did not investigate the underlying molecular mechanism by which MSU crystals promote TTP expression. It has been reported that IL-1β, TNF-α, IL-6, COX-2 and iNOS are targets of TTP-induced mRNA degradation (34, 35), and these inflammatory factors are also involved in MSU crystal-induced inflammation. Our data indicated that TTP knockdown led to elevated mRNA and protein levels of these inflammatory factors in macrophages stimulated by MSU crystals. The NLRP3 inflammasome is the main pathway of MSU crystal triggering cell inflammatory response. Haneklaus et al. reported that TTP is a crucial negative regulator of the NLRP3 inflammasome (17), and consistent with this report, we also observed that TTP knockdown promoted NLRP3 expression at the posttranscriptional level and NLRP3 inflammasome activation induced by MSU crystals.

PP2A is a phosphatase that can dephosphorylate TTP (36) and is closely associated with TTP activation. To date, the best known PP2A activator is the sphingosine analog FTY720, FTY720 has other roles, most notably as a functional antagonist of the S1P pathway. Previous study has shown that the active metabolites of FTY720 can activate PP2A and inhibit the production of IL-1β induced by MSU crystals (37), but the molecular mechanism of its inhibition of IL-1β production has not been thoroughly explored. Arctigenin is a natural product, so we chose to study the effect of ATG on the inflammatory response induced by MSU crystals and its molecular mechanism. This study showed that MSU crystals promoted the activity of PP2A and that PP2A agonist inhibited the inflammation induced by MSU crystals *in vitro* and *in vivo*. Previous studies have reported that Arctigenin can alleviate the activity of NLRP3 inflammasome (38, 39). Our data showed that Arctigenin augmented PP2A activity, ameliorated inflammatory factors expression and NLRP3 inflammasome activation in J774A.1 cells stimulated by MSU crystals. More importantly, Arctigenin may mediate its anti-inflammatory effects in a TTP-dependent manner. Mitochondrial dysfunction is considered to be an important factor triggering the activation of the NLRP3 inflammasome (38), and mtROS overproduction is a key factor in NLRP3 inflammasome activation  (39). It has been reported that Arctigenin can exert antioxidative effects and enhance the activities of antioxidant enzymes (21). We found that MSU crystal treatment led to a significant increase in intracellular and mitochondrial ROS, and Arctigenin treatment reduced the ROS production induced by MSU crystals, suggesting that the mechanism by which Arctigenin inhibits the activation of the NLRP3 inflammasome may be related to the reduction of ROS production. Arctigenin-mediated inhibition of mitochondrial ROS production is beneficial for improving mitochondrial function. Mitochondrial dysfunction is closely related to the loss of mitochondrial membrane potential. In this study, we found that Arctigenin inhibited the decline in mitochondrial membrane potential. Our study for the first time shows that TTP plays an important role in regulating mitochondrial dysfunction induced by MSU crystals and ATG regulates mitochondrial dysfunction in a TTP-dependent way, which is of great significance for further studies on the anti-inflammatory molecular mechanisms of ATG and TTP. It has been reported that ROS can regulate lysosomal membrane stability (40). In our study, Arctigenin also relieved lysosomal membrane stability, which is a crucial upstream regulatory factor for NLRP3 activation (41). More importantly, TTP knockdown impeded the protective function of ATG on lysosomes. These data suggest that Arctigenin may protect lysosomal membrane stability by alleviating mitochondrial oxidative stress in a TTP-dependent manner.

As the NLRP3 inflammasome is the main pathway in the response to MSU crystals, strategies that inhibit its activation or affect its activity can relieve gouty inflammation. Although TTP has been reported to play an important role in many inflammatory responses, we investigated that TTP could not only modulate the expression of inflammation-related genes, but also regulate the cleavage of caspase-1 in NLRP3 inflammasome in MSU crystal-induced inflammation, which may have an important influence on the pathogenesis of GA. Treatment with Arctigenin effectively inhibited the inflammatory response induced by MSU crystals in animal models of peritoneal inflammation and arthritis. Our data indicated that Arctigenin could both improve mitochondrial function and promote autophagy flux in a TTP-dependent manner; it would be interesting to further investigate how Arctigenin regulates the relationship between mitochondrial autophagy induced by MSU crystals and the activation of NLRP3 inflammasome.

**Figure 9:**
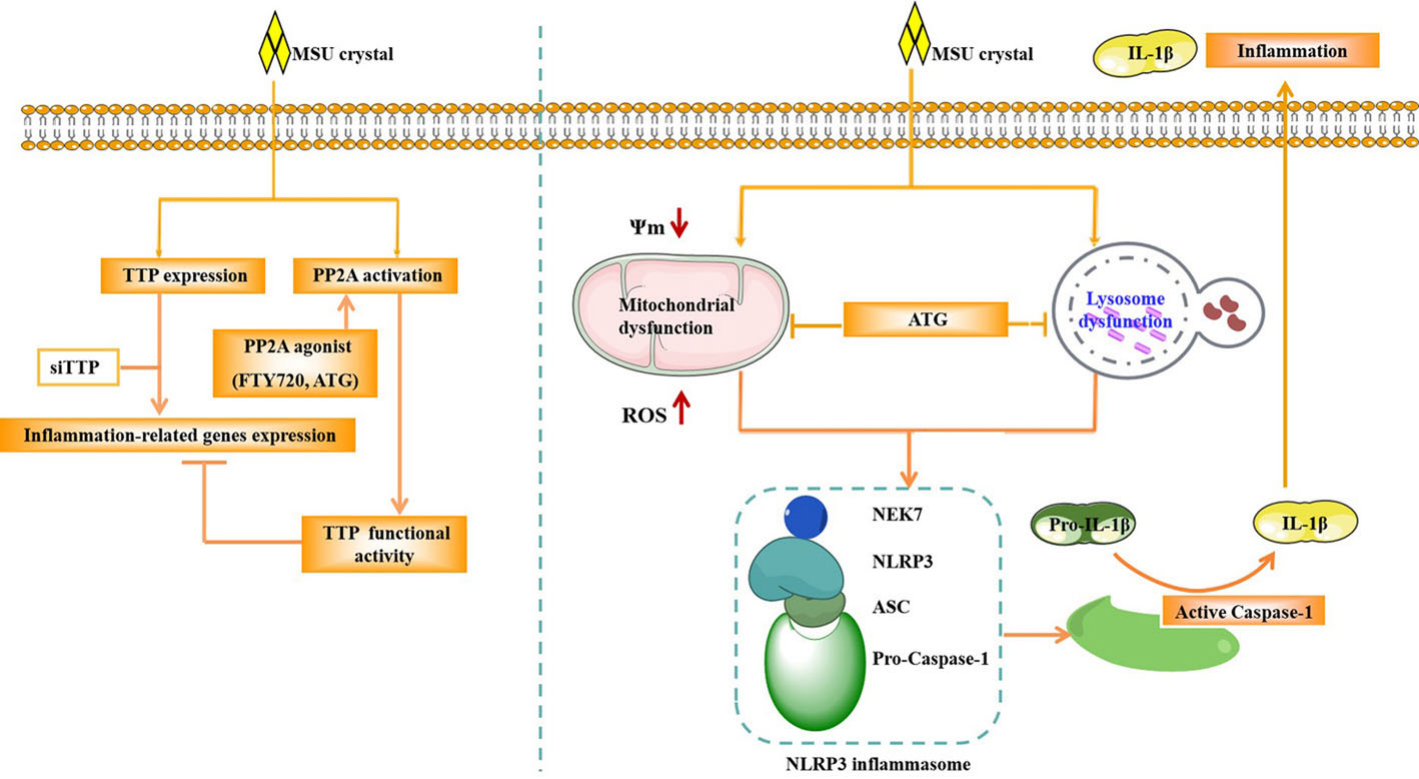
Schematic of the signaling pathway involved in TTP and PP2A activity in the MSU crystal-induced inflammatory response. After MSU crystal stimulation, TTP expression and PP2A activity were increased. TTP knockdown promoted the expression of inflammation-related genes induced by MSU crystals. However, PP2A agonist (ATG) treatment relieved the expression of inflammation-related genes induced by MSU crystals. ATG may alleviate the production of mitochondrial reactive oxygen species, increase mitochondrial membrane potential and improve lysosomal function, thereby inhibiting the activity of NLRP3 inflammasome, and ultimately reducing the inflammation caused by MSU crystals.

In Conclusion, our study demonstrated that TTP can regulate the expression of inflammation-related gene and the activity of NLRP3 inflammasome in response to MSU crystals (**Figure 9**). PP2A agonist can mitigate inflammatory response induced by MSU crystals through by improving lysosome and mitochondrial function, thereby inhibiting the activation of NLRP3 inflammasomes (**Figure 9**). The anti-inflammatory function of the PP2A agonist (Arctigenin) might be associated with TTP activation in MSU crystal-induced inflammation.

## Data Availability Statement

The original contributions presented in the study are included in the article/**Supplementary Material**. Further inquiries can be directed to the corresponding author.

## Ethics Statement

The animal study was reviewed and approved by Ethics Committee of North Sichuan Medical College.

## Author Contributions

YX and MZ initiated and designed this study. FC and HJ performed and analyzed the majority of the experiments. LL, TQ, and QH wrote the manuscript. FL and LR performed and analyzed the individual experiments. JL and YX performed data curation. MZ supervised the study. All authors contributed to the article and approved the submitted version.

## Funding

This research was supported by grants (No. 81972119) from the National Natural Science Foundation of China; the Sichuan Province Science and Technology Support Project (2018JY0158); and the Nanchong City Science and Technology Support Project (19SXHZ0456 and 19YFZJ0045).

## Conflict of Interest

The authors declare that the research was conducted in the absence of any commercial or financial relationships that could be construed as a potential conflict of interest.

## Abbreviations

TTP, Tristetraprolin; MSU, monosodium urate; ATG, Arctigenin; RA, rheumatoid arthritis; PP2A, Protein phosphatase 2A; ROS, reactive oxygen species; SOD, superoxide dismutase; CAT, catalase; GPX, glutathione peroxidase; MtROS, mitochondrial reactive oxygen species; MMP, mitochondrial membrane potential; MPO, myeloperoxidase; HC, healthy control; AGA, acute gouty arthritis; IGA, intercritical gouty arthritis; Ctsb, cathepsin B; Ctsd, cathepsin D; iNOS, induce nitric oxide synthetase; NLRP3, NOD-like receptor family, pyrin domain containing 3; TNF-α, tumor necrosis factor α; TFEB, transcription factor EB; H&E, hematoxylin and eosin; IL-1β, interleukin 1 beta; PBMCs, Peripheral blood mononuclear cells; DHE, Dihydroethidium.
