# Supplementary material for: Targeting Tristetraprolin Expression or Functional Activity Regulates Inflammatory Response Induced by MSU Crystals
Source: Front Immunol. 2021 Jul 16;12:675534. doi: 10.3389/fimmu.2021.675534 (PMC8322984; doi:10.3389/fimmu.2021.675534)
Supplement: Supplementary file 1 [file DataSheet_1.doc]

**Details of the antibodies used in the Western blotting and immunofluorescence**

Western blotting was performed using the following antibodies: anti-TTP (LS-B1572, LSBio), anti-LC3A/B(D3U4C, CST), anti-P62(382862, ZENBIO, Chengdu, China), anti-TFEB(A303-673A-M, BETHYL), anti-Lamin B1(340569, ZENBIO, Chengdu, China), anti-Phospho-MK2 (phospho T334, ab131504, Abcam), anti-Phospho-ERK1 (phospho T202, ab194776, Abcam), anti-COX-2 (ET1601-23, HUABIO, Hangzhou, China), anti-iNOS (ER1706-89, HUABIO, Hangzhou, China), anti-NLRP3 (ET1706-72, HUABIO, Hangzhou, China), anti-IL-1β (Asp117, CST), anti-Caspase-1 (ET1608-69, HUABIO, Hangzhou, China), anti-MPO (208670, Abcam), anti-β-Actin (M3873, BosterBio, Wuhan, China), anti-α-Tubulin (ET1602-4, HUABIO, Hangzhou, China), anti-GAPDH (ET1601-4, HUABIO, Hangzhou, China), Vinculin (BA2934, BosterBio, Wuhan, China).

The following antibodies are used for immunofluorescence: anti-TTP (LS-B1572, LSBio), anti-MPO (208670, Abcam), anti-Ly-6G (0809-11, HUABIO, Hangzhou, China), anti-CD11b (BM3925, BosterBio, Wuhan, China).

**Supplementary Figures**

**
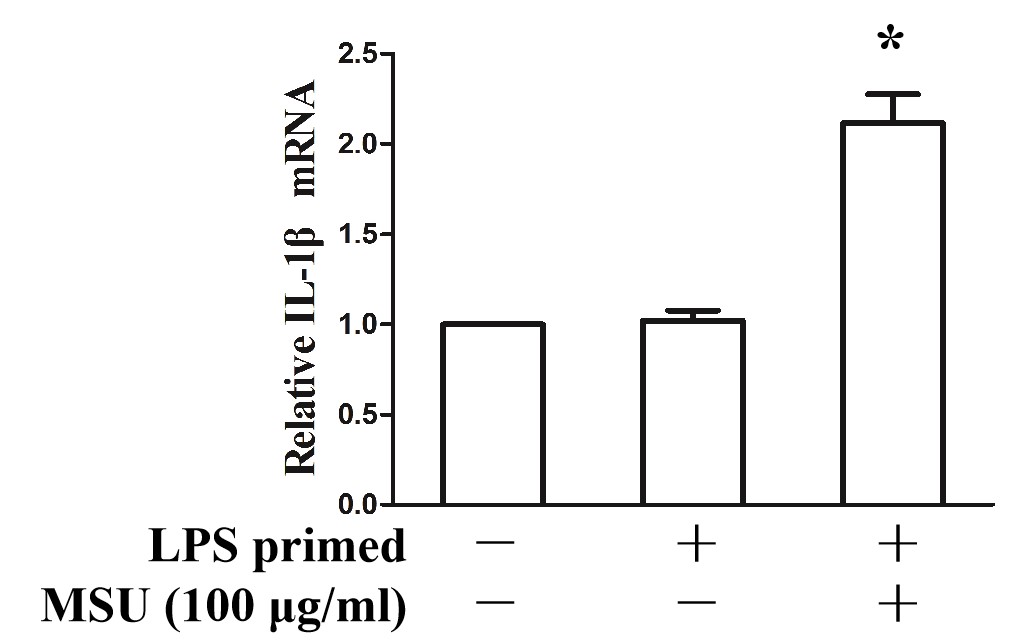
**

**Supp Figure 1** J774A.1 cells were primed with 100 ng/ml LPS and then stimulated with MSU crystals (50 μg/ml) for 9 h. IL-1β mRNA level was detected.


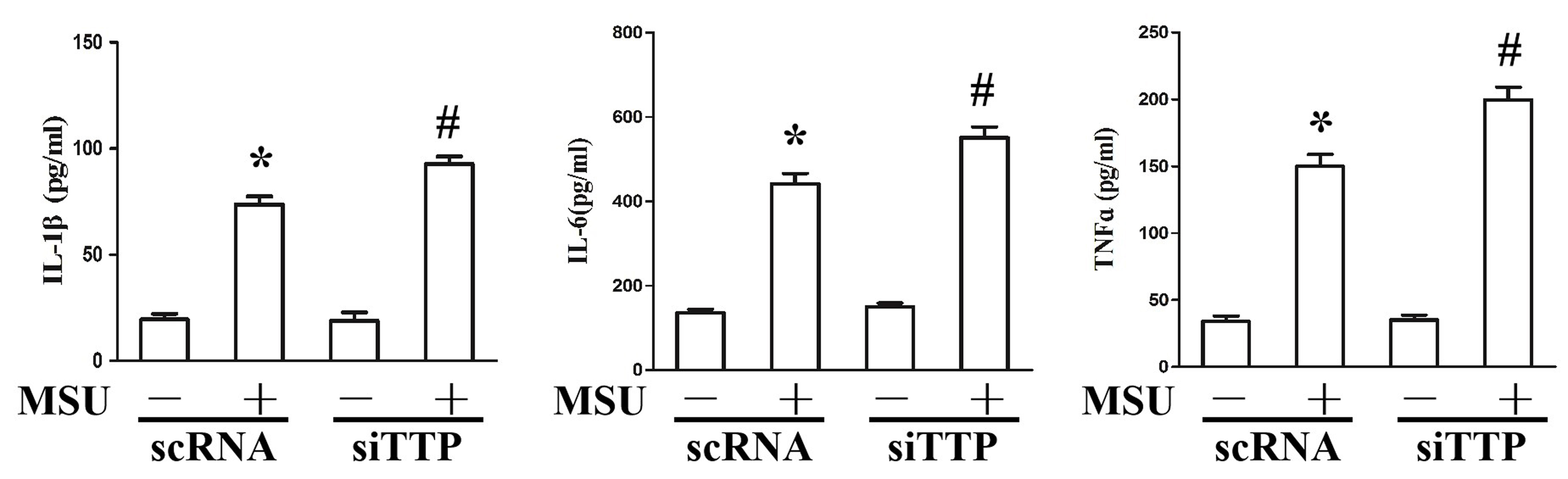


**Supp Figure 2** TTP knockdown increased the secretion of IL-1β, TNF-α and IL-6 in MSU crystal-induced J774A.1 cells.

J774A.1 cells were transfected with TTP-targeted siRNA (siTTP) or scramble RNA (scRNA) for 48 h, primed with 100 ng/ml LPS and then stimulated with MSU crystals (50 μg/ml) for 9 h. IL-1β, TNF-α and IL-6 secretion was detected. All the data are expressed as the means ± SEM from n=3 independent experiments, * P < 0.05 vs. without MSU crystals treatment; **^#^** P < 0.05 vs. scRNA transfection + MSU crystals treatment.


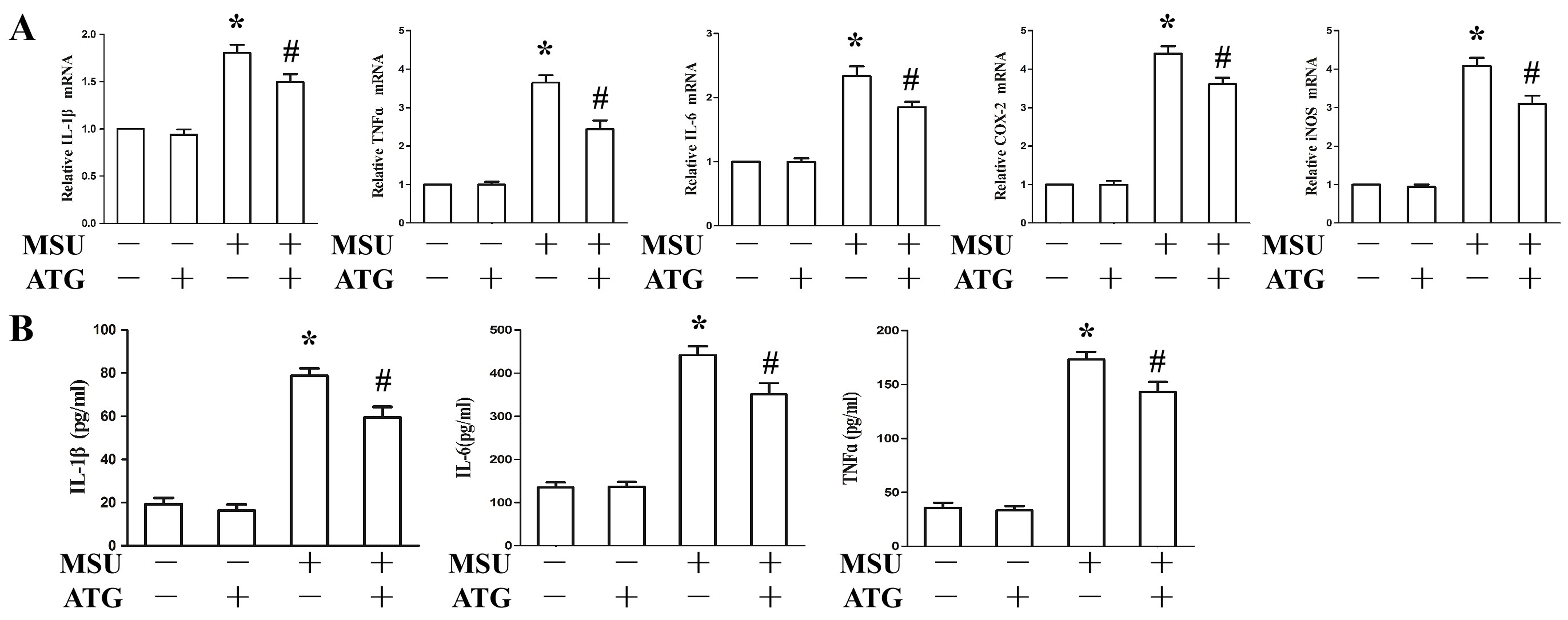


**Supp Figure 3** ATG affected the secretion of IL-1β, TNF-α and IL-6 in MSU crystal-induced J774A.1 cells

J774A.1 cells were pretreated with ATG (5 μM) for 1 h, primed with 100 ng/ml LPS and then treated with MSU crystals (50 μg/ml) for 9 h. **(A)** The total RNA was extracted and cell culture supernatants was collected for further experiments. **(B)** The secretion of IL-1β, TNF-α and IL-6 was determined using ELISA in the culture supernatants after ATG administration. All the data are expressed as the means ± SEM from n=3 independent experiments, * P < 0.05 vs. without MSU crystals treatment; **^#^** P < 0.05 vs. MSU crystals treatment +vehicle.


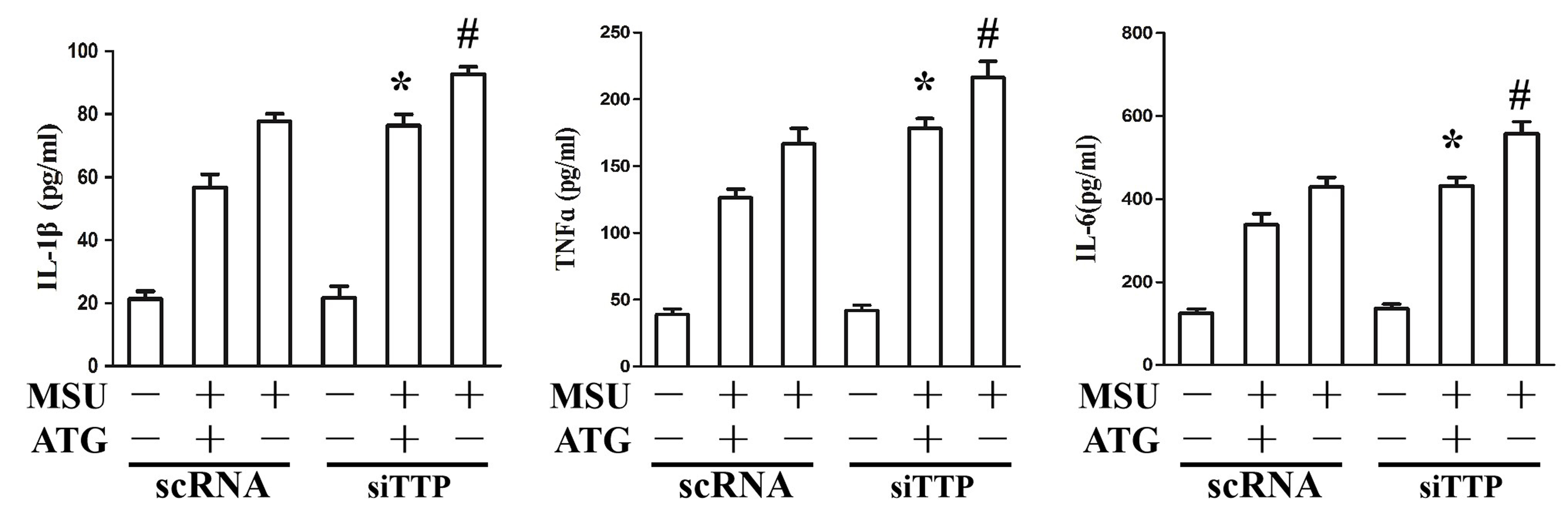


**Supp Figure 4** TTP knockdown reverses ATG repression of MSU crystal-induced IL-1β, TNF-α and IL-6 secretion.

J774A.1 cells were either transfected with scRNA or siTTP for 48 h, treated with 5 μM ATG for 1 h, primed with LPS for 1 h, and then stimulated with MSU crystals (50 μg/ml) for 9 h. IL-1β, TNF-α and IL-6 release in the cell culture supernatants was measured using ELISA. All the data are expressed as the means ± SEM from n=3 independent experiments, * P < 0.05 vs. MSU crystals treatment + ATG + scRNA; **^#^** P < 0.05 vs. MSU crystals treatment + vehicle + siTTP.


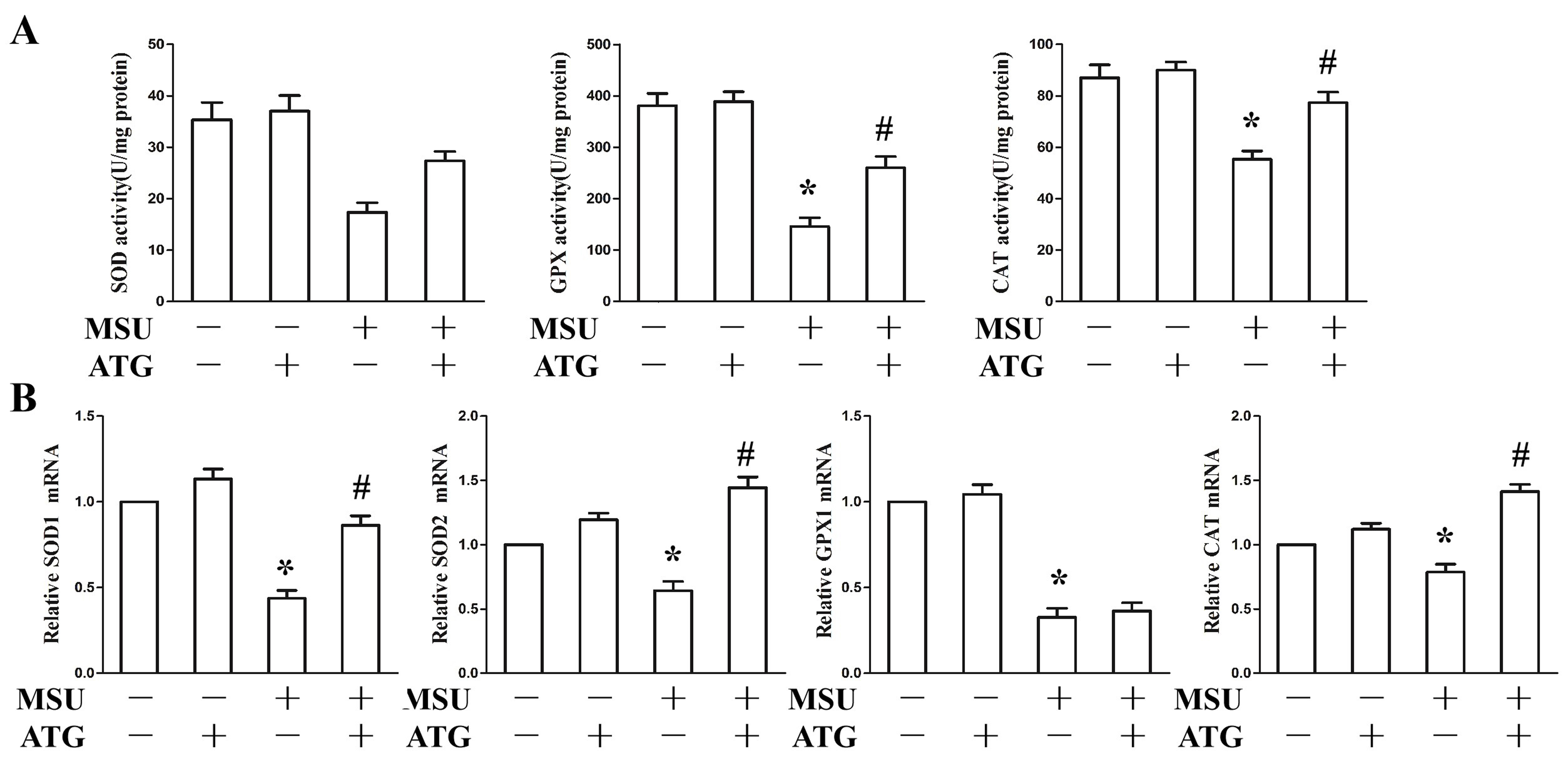


**Supp Figure 5** Effects of ATG treatment on the MSU crystal-induced antioxidant enzymes activity and mRNA levels of antioxidant enzymes.

**(A, B)** J774A.1 cells were pretreated with ATG (5 μM) for 1 h, primed with LPS for 1 h and then treated with MSU crystals (50 μg/ml) for 9 h. **(A)** The activities of SOD, GPX, and CAT. **(B)** The mRNA levels of SOD1, SOD2, GPX1 and CAT, respectively. All the data are expressed as the means ± SEM from n=3 independent experiments, * P < 0.05 vs. without MSU crystals treatment; **^#^** P < 0.05 vs. MSU crystals treatment +vehicle.


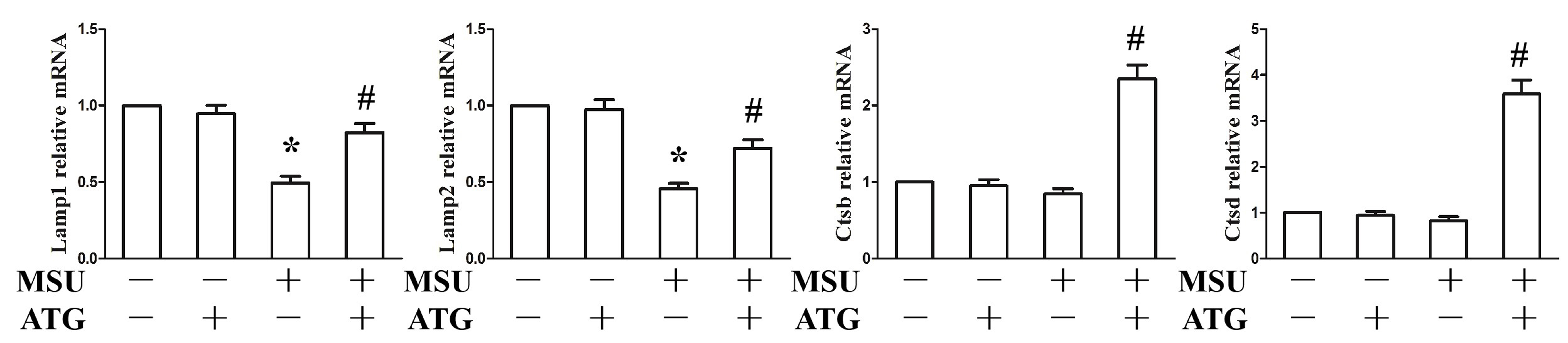


**Supp Figure 6** Effects of ATG on the mRNA expression of Lamp1, Lamp2, Ctsb, and Ctsd.

J774A.1 cells were either transfected with scRNA or siTTP for 48 h, pretreated with 5 μM of ATG for 1 h , primed with LPS for 1 h, then stimulated with MSU crystals (50 μg/ml) for 9 h. The mRNA levels of Lamp1, Lamp2, Ctsb, and Ctsd were determined by RT-PCR. All the data are expressed as the means ± SEM from n=3 independent experiments, * P < 0.05 vs. without MSU crystals treatment; **^#^** P < 0.05 vs. MSU crystals treatment +vehicle.


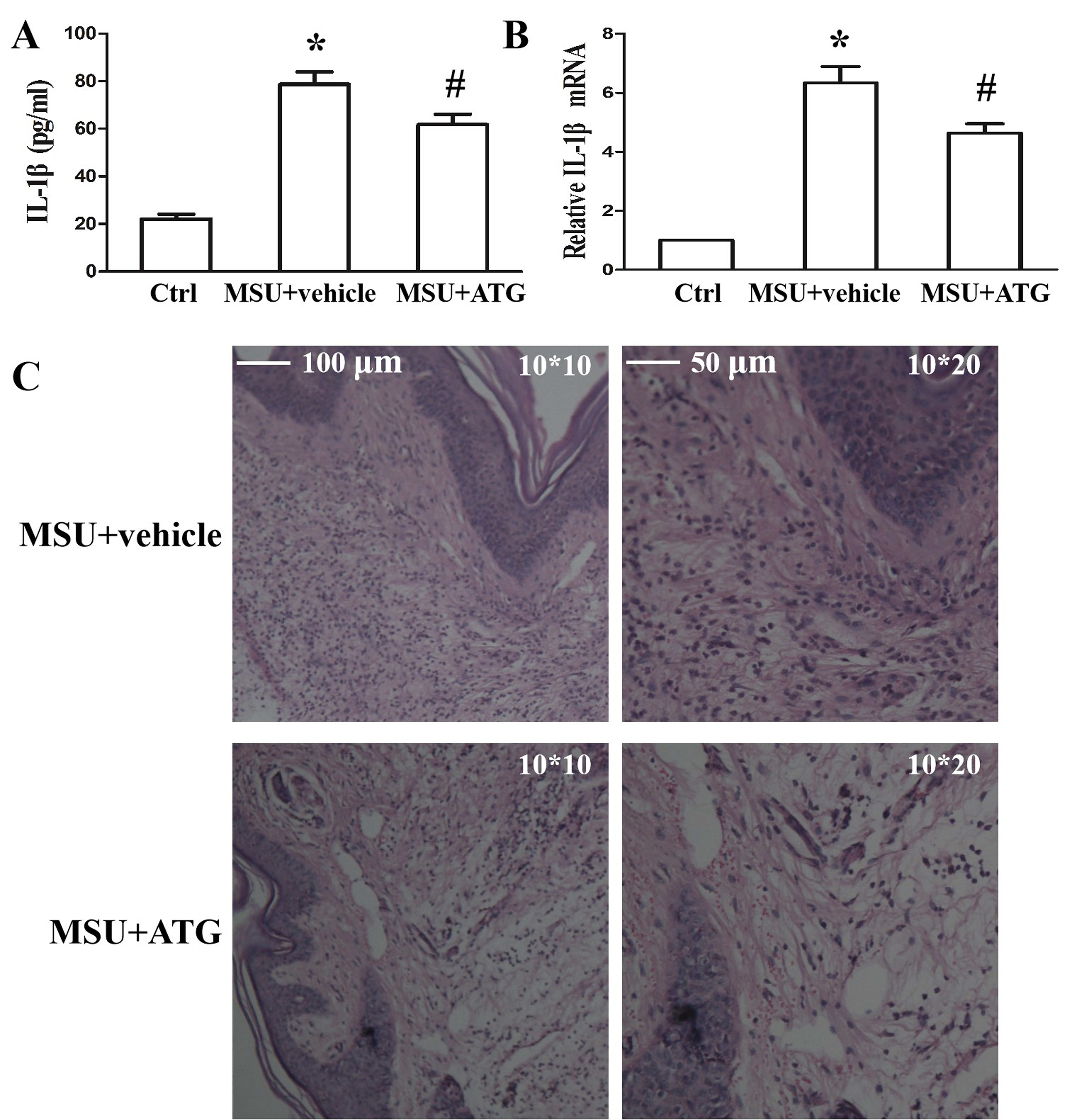


**Supp Figure 7** Effects of ATG on MSU crystal-induced IL-1β expression and inflammatory cell infiltration in mouse model of peritonitis or arthritis.

**(A)** Supernatants of peritoneal fluid were analyzed by ELISA for IL-1β. **(B)** IL-1β mRNA levels in foot pad tissue. **(C)** HE staining of foot pad tissue section. n=5 for each group, * P < 0.05 vs. Ctrl; **^#^** P < 0.05 vs. MSU crystals + vehicle. All the data are expressed as the means ± SEM from n=3 independent experiments.


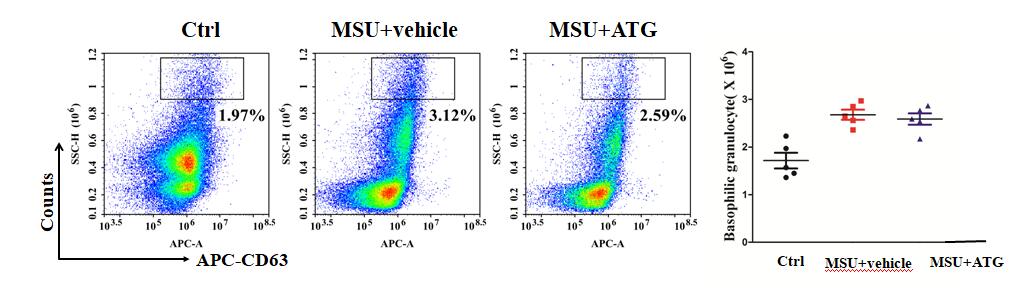


**Supp Figure 8** Effects of ATG on MSU crystal-induced basophilic granulocyte infiltration in a mouse model of peritonitis.

After the cells precipitated from the peritoneal fluid and then were stained with APC-CD63 Ab. The percentage and cell numbers of basophilic granulocyte were analyzed using FACS.
